# Intensive Teenage Activity Is Associated With Greater Muscle Hyperintensity on T1W Magnetic Resonance Imaging in Adults With Dysferlinopathy

**DOI:** 10.3389/fneur.2020.613446

**Published:** 2020-12-16

**Authors:** Ursula Moore, Marni Jacobs, Roberto Fernandez-Torron, Jaume LLauger Rossello, Fiona E. Smith, Meredith James, Anna Mayhew, Laura Rufibach, Pierre G. Carlier, Andrew M. Blamire, John W. Day, Kristi J. Jones, Diana X. Bharucha-Goebel, Emmanuelle Salort-Campana, Alan Pestronk, Maggie C. Walter, Carmen Paradas, Tanya Stojkovic, Madoka Mori-Yoshimura, Elena Bravver, Elena Pegoraro, Jerry R. Mendell, Kate Bushby, Volker Straub, Jordi Diaz-Manera

**Affiliations:** ^1^The John Walton Muscular Dystrophy Research Centre, Translational and Clinical Research Institute, Newcastle University, Newcastle Hospitals NHS Foundation Trust, Newcastle upon Tyne, United Kingdom; ^2^Division of Biostatistics and Study Methodology, Center for Translational Science, Children's National Health System, Washington, DC, United States; ^3^Pediatrics, Epidemiology and Biostatistics, George Washington University, Washington, DC, United States; ^4^Neuromuscular Area, Biodonostia Health Research Institute, Neurology Service, Donostia University Hospital, Donostia-San Sebastian, Spain; ^5^Radiology Department, Hospital de la Santa Creu i Sant Pau, Universitat Autònoma de Barcelona, Barcelona, Spain; ^6^Magnetic Resonance Centre, Translational and Clinical Research Institute, Newcastle University, Newcastle upon Tyne, United Kingdom; ^7^Jain Foundation, Newcastle upon Tyne, Seattle, WA, United States; ^8^Université Paris-Saclay, CEA, DRF, Service Hospitalier Frederic Joliot, Orsay, France; ^9^Department of Neurology and Neurological Sciences, Stanford University School of Medicine, Stanford, CA, United States; ^10^The Children's Hospital at Westmead, The University of Sydney, Sydney, NSW, Australia; ^11^Department of Neurology Children's National Health System, Washington, DC, United States; ^12^National Institutes of Health (NINDS), Bethesda, MD, United States; ^13^Service des maladies neuromusculaire et de la SLA, Hôpital de La Timone, Marseille, France; ^14^Department of Neurology Washington University School of Medicine, St. Louis, MO, United States; ^15^Department of Neurology, Friedrich-Baur-Institute, Ludwig-Maximilians-University of Munich, Munich, Germany; ^16^Center for Biomedical Network Research on Eurodegenerative Diseases, Instituto de Salud Carlos III, Madrid, Spain; ^17^Neuromuscular Unit, Department of Neurology, Hospital U. Virgen del Rocío/Instituto de Biomedicina de Sevilla, Sevilla, Spain; ^18^Centre de référence des maladies neuromusculaires, Institut de Myologie, AP-HP, Sorbonne Université, Hôpital Pitié-Salpêtrière, Paris, France; ^19^Department of Neurology, National Center Hospital, National Center of Neurology and Psychiatry Tokyo, Tokyo, Japan; ^20^Carolinas Neuromuscular/ALS-MDA Center, Neuroscience Institute, Carolinas HealthCare System, Charlotte, NC, United States; ^21^Department of Neuroscience, University of Padova, Padova, Italy; ^22^The Abigail Wexner Research Institute at Nationwide Children's Hospital, Columbus, OH, United States; ^23^Neuromuscular disorders Unit, Department of Neurology, Hospital de la Santa Creu I Sant Pau, Barcelona, Spain; ^24^Centro de Investigación Biomédica en Red en Enfermedades Raras (CIBERER), Valencia, Spain

**Keywords:** dysferlinopathy, Magnetic Resonace Imaging (MRI), Miyoshi myopathy, LGMDR2, limb girdle muscle dystrophy, exercise

## Abstract

Practice of sports during childhood or adolescence correlates with an earlier onset and more rapidly progressing phenotype in dysferlinopathies. To determine if this correlation relates to greater muscle pathology that persists into adulthood, we investigated the effect of exercise on the degree of muscle fatty replacement measured using muscle MRI. We reviewed pelvic, thigh and leg T1W MRI scans from 160 patients with genetically confirmed dysferlinopathy from the Jain Foundation International clinical outcomes study in dysferlinopathy. Two independent assessors used the Lamminen-Mercuri visual scale to score degree of fat replacement in each muscle. Exercise intensity for each individual was defined as no activity, minimal, moderate, or intensive activity by using metabolic equivalents and patient reported frequency of sports undertaken between the ages of 10 and 18. We used ANCOVA and linear modeling to compare the mean Lamminen-Mercuri score for the pelvis, thigh, and leg between exercise groups, controlling for age at assessment and symptom duration. Intensive exercisers showed greater fatty replacement in the muscles of the pelvis than moderate exercisers, but no significant differences of the thigh or leg. Within the pelvis, Psoas was the muscle most strongly associated with this exercise effect. In patients with a short symptom duration of <15 years there was a trend toward greater fatty replacement in the muscles of the thigh. These findings define key muscles involved in the exercise-phenotype effect that has previously been observed only clinically in dysferlinopathy and support recommendations that pre-symptomatic patients should avoid very intensive exercise.

## Introduction

Dysferlinopathy is an autosomal recessively inherited form of muscular dystrophy caused by mutations in the *DYSF* gene. It usually presents in early adulthood and is characterized by progressive weakness and wasting of skeletal muscles (1).

Unlike many other forms of muscular dystrophy, patients with dysferlinopathy often report performing a large amount of physical activity as children and young adults, before their symptoms first present (2, 3). This intensive exercise appears to be detrimental and we have previously demonstrated that higher levels of exercise before symptom onset is associated with both earlier patient reported symptom onset and earlier subsequent wheelchair requirement (4).

The association of exercise and disease progression in dysferlinopathy has been investigated in mouse models, with interesting results. Eccentric muscle contractions (such as running) cause more rapid progression of muscular dystrophy pathology and greater functional weakness, while concentric muscle contraction (such as swimming) appeared to be protective against both general disease progression and in mitigating the myofiber damage caused by subsequent eccentric muscle contraction (5, 6). This finding clearly has implications for patients in terms of the type or intensity of exercise that should be recommended. However, murine models of dysferlinopathy are not particularly good mimics of the human phenotype, being much less severe, and it is not certain if these findings can be generalized (6). In humans with dysferlinopathy, pathological differences depending on exercise type or intensity have not been assessed and it is not clear if early influences of exercise would remain detectable after many years of symptomatic disease.

Magnetic resonance imaging (MRI) can be used to non-invasively and objectively measure increases in intramuscular fat, acting as marker for muscle pathology. T1W sequences clearly delineate fat and muscle and are used to produce images for visual inspection to determine the degree of the muscle that has been replaced by fat. This method is used to determine both the pattern and severity of muscle involvement in neuromuscular disorders, and is widely used to support genetic diagnosis (7, 8). T1W MRI has been used to characterize a distinctive pattern of muscle involvement in dysferlinopathy, with certain muscles consistently demonstrating greater, or earlier, fat replacement than others (9).

In this study we use T1W images from this previously described cohort, in combination with questionnaire derived information about pre-symptomatic activity levels to objectively assess the impact of teenage exercise on muscle fat replacement. We review if this supports the patient reported earlier onset and more rapid loss of ambulation in intensive exercisers, if exercise has differential effects in muscle, such that some muscles are more impacted than others and if conclusions can be reached about the types of exercise that may be detrimental in dysferlinopathy.

## Methods

### Study Subjects

This investigation reviews MRI imaging from the Jain Foundation's international longitudinal *Clinical Outcomes Study for Dysferlinopathy* (COS). This study included 201 patients with genetically confirmed dysferlinopathy from 15 sites internationally (10). This study received ethical approval in all participating countries. MRI was not mandatory for inclusion in the study and some sites could only perform lower limb MRI (not including the pelvis). Overall, 182 patients had a baseline MRI, including 84 patients with a whole body scan and 98 patients with only lower limb scans. Imaging from 22 patients did not produce adequate images for visual scoring to be applied (see below) and were excluded. This investigation therefore reviews MRI imaging from 160 patients.

This report uses the questionnaire based exercise information (Supplementary Material) and the MRI images collected from the patients screening and baseline visits, respectively. These visits also involved medical interview and examination, physiotherapy assessment, blood sampling, cardiac, and respiratory investigations. A cross-sectional description of the baseline cohort demographic and functional information (10), and pattern of muscle involvement on T1W MRI at baseline (9) have been previously published.

### Semiquantitative MRI Assessment

Semiquantitative assessments of MRIs were performed by a blinded neurologist (RF-T) and radiologist (JL), who independently evaluated axial T1-weighted sequences with the Lamminen-Mercuri visual scale, with an inter-rater agreement kappa of 0.93 (95% CI 0.91–0.96), as previously reported (9). For the 4% of scans where observers scores did not match, observers reviewed the muscles together and agreed a final score (9). All 160 patients had complete Lamminen-Mercuri score results for the lower leg muscles—Tibialis anterior, extensor digitorum, peroneus longus, peroneus brevis, gastrocnemius medialis and lateralis, soleus, flexor digitorum, and tibialis posterior. Complete imaging of adductors was not included in some patients, leaving 106 of these 160 patients who also had a complete set of Lamminen-Mercuri scores for thigh muscles (Adductor brevis, longus an major, quadratus femoris, rectus femoris, vastus lateralis, vastus intermedius and vastus medialis, sartorius, gracilis, semimembranosus, semitendinosus, long, and short head of biceps femoris). Full pelvic muscle Lamminen-Mercuri scores were available for 67 of the 160. Pelvic muscles were psoas, pectineus, piriformis, iliacus, gluteus medius, gluteus maximus, gluteus minor, tensor fasciae latae, obturator internus, and obturator externus.

### Exercise Scoring

At the screening visit, patients reported the type and frequency of all regular activities performed as children and teenagers (Supplementary Material). Exercise was coded based on the maximum patient reported frequency between the age of 10 and 18 years, and the metabolic equivalent (MET) of the activities described, as previously reported (4). Group 0 reported no physical activity, group 1 reported vigorous activity occasionally/monthly, or moderate activity once a week, group 2 reported moderate activity multiple times a week or vigorous activity once weekly, and group 3 reported vigorous activity multiple times per week.

### Statistical Analysis

Age at MRI and symptom duration were not normally distributed and so were compared in a stepwise fashion between exercise groups using the Wilcoxon-Mann-Whitney test for non-parametric unpaired samples (i.e., group 0 vs. group 1, group 0 vs. group 2 and so forth for comparison between all groups).

A mean Lamminen-Mercuri score for the distal leg was calculated from the sum of scores of each individual muscle (*n* = 160). This was repeated to generate a mean Lamminen-Mercuri score for the thigh (*n* = 106) and the pelvis (*n* = 67). Muscles included are listed above.

To determine if there was any difference in Lamminen-Mercuri scores between exercise groups, mean Lamminen-Mercuri scores of the a. leg, b. thigh and c. pelvis were compared between exercise groups using type III ANCOVA, using the “Anova” function from the package “car” in the programme R. Age at MRI and symptom duration were assessed as covariates. Firstly, age was considered as a covariate to determine if patients in a particular exercise group at any given age had a different Lamminen-Mercuri score than those in another group. Secondly, symptom duration and age were combined as covariates to determine if fat replacement on MRI progressed more rapidly (from the onset of symptoms) in one exercise group than another. *Post-hoc* analysis of significant (*p* < 0.05) ANCOVA results was completed using the “lsmeans()” function, from the package “lsmeans.” This was to identify which exercise groups were significantly different from each other, and thus causing the significant ANCOVA result.

For visual representation of the exercise dependent effects, linear modeling was used. A linear model of Lamminen-Mercuri score was generated with age and exercise group as covariates. This allowed calculation of the mean model predicted Lamminen-Mercuri score for each exercise group as if all patients were of the same age (39 years—the mean age of the cohort). This was repeated with disease duration as an additional covariate (along with age) to produce model predicted Lamminen-Mercuri scores as if all patients had had the same disease duration (17 years—the mean disease duration of the cohort) and age.

Muscle groups showing significant differences between exercise groups were then further reviewed with additional ANCOVA of the Lamminen-Mercuri scores of the individual muscles within that group and compared in the same way as for the muscle groups.

In order to review if results differed in a subset of patients with a shorter disease duration, we repeated this analysis of pelvic, thigh and leg muscle groups in patients with symptoms for <15 years at the time of assessment.

*Post-hoc* review of *p*-values using Bonferonni correction for multiple comparisons was performed. No power calculations were conducted to determine group size as data was from an existing study.

## Results

### Demographics

Patient reported age of symptom onset was later in the inactive group 0 than in all of the other exercise groups (Table 1). Patients in group 0 were significantly older at the time of assessment than those in the extremely active group 3, who were in turn older than those in group 2 (*p* < 0.05) (Table 1). Overall, patients in the moderately active group 2 had a shorter median symptom duration at the time of assessment than those in group 0 or in group 3 (*p* < 0.05). Number of patients in group 1 was small (17 patients in distal leg MRI group) with variable age and disease duration, which was not significantly different from those in other exercise groups.

**Table 1:** Table showing demographic factors of patients in each exercise group at baseline.

	**Exercise group**			
	**0**	**1**	**2**	**3**
**Distal leg MRI (*****n*** **= 160)**				
Number of patients	39	17	54	50
Male (%)	14 (36)	9 (53)	30 (56)	28 (56)
Symptom onset age in years—median (range)^*^	25.00 (12–48)	17.00 (12–60)	18.00 (10–41)	18.00 (12.5–40)
Symptom duration in years—median (range)^**^	20.00 (4–41)	11.00 (2–42)	12.50 (2–38)	17.00 (3–51)
Age at assessment in years—median (range)^***^	47.00 (22–86)	35.00 (15–71)	31.50 (15–57)	37.50 (22–67)
**Thigh MRI in (*****n*** **= 106)**
Number of patients	25	12	40	29
Male (%)	9 (36)	6 (50)	23 (58)	17 (59)
Symptom onset age in years – median (range)(^*^)	25.00 (12–48)	18.5 (12–60)	18.00 (10–41)	17.5 (13–28)
Symptom duration in years—median (range)	21(4–41)	18.5 (3–42)	13.00 (2–38)	16.5 (3–51)
Age at assessment in years—median (range)^****^	47.00 (32–86)	37.5 (15–71)	30.00 (15–57)	37.00 (22–64)
**Pelvic MRI (*****n*** **= 67)**				
Number of patients	11	6	28	22
Male (%)	2 (18)	3 (50)	13 (46)	12 (55)
Symptom onset age in years—median (range)^*^	26.00 (14–39)	16.00 (12–60)	18.00 (12–41)	17.50 (13–27)
Symptom duration in years—median (range)^**^	16.00 (10–41)	14.00 (3–26)	13.00 (4–23)	19.00 (6–51)
Age at assessment in years—median (range)^***^	47.00 (32–66)	32.50 (15–71)	32.500 (19–57)	39.00 (22–64)

*MRI: T1 weighted magnetic resonance imaging*.

* *Median age of symptom onset significantly higher in group 0 compared to group 1, 2, and 3*.

** *Median disease duration significantly shorter in group 2 than in group 0 or group 3*.

*** *Group 0 are significantly older than group 3 who are, in turn, significantly older than group 2*.

**** *Group 0 is significantly older than group 2*.

In the groups of patients who had pelvic or thigh imaging, the median age of onset, age at assessment and symptom duration in each exercise group did not differ significantly from that in the larger cohort.

### Age Adjusted Mercuri Score

**Figure 1:**
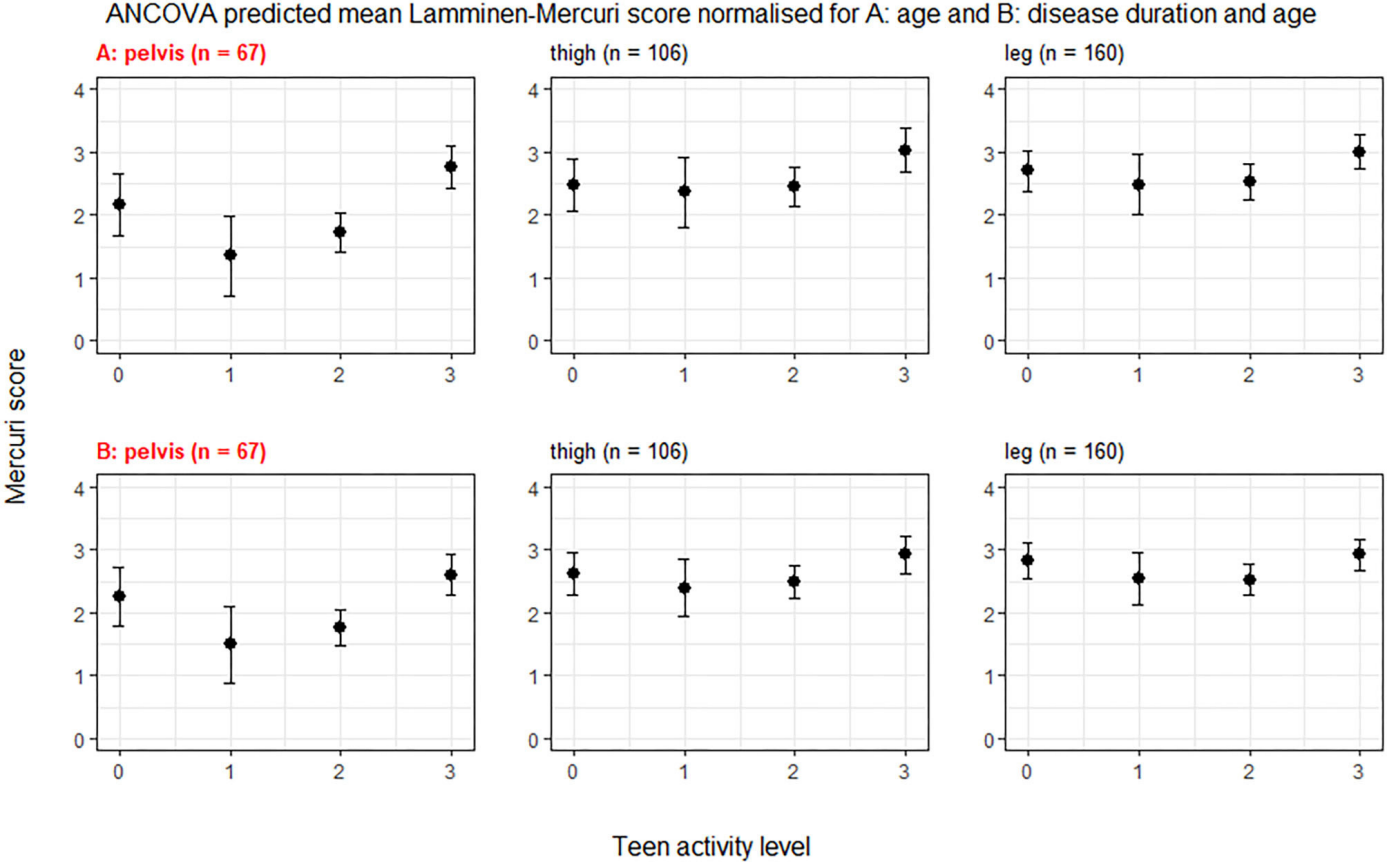
ANCOVA predicted Lamminen-Mercuri scores for each exercise group. The top row **(A)** shows predicted score in each exercise group (0, 1, 2, or 3) adjusted for age—i.e., as though all patients were the cohort mean age of 39 years. The bottom row **(B)** shows predicted score in each exercise group (0, 1, 2, or 3) adjusted for disease duration and age—i.e., as though all patients had an age of 39 years and the cohort mean symptom duration of 17 years. Bars show the 95% confidence interval. Red text denotes groups showing significant differences on ANCOVA (*p* < 0.005).

There were significant differences between exercise groups in the mean predicted Lamminen-Mercuri score of the pelvic muscles (*p* ≤ 0.0001), when controlling for age as a covariate. This was driven by a significantly higher predicted Lamminen-Mercuri score in exercise group 3, compared to group 1 (3 vs. 1, *p* = 0.0018) and group 2 (3 vs. 2, *p* = 0.0002). There was no significant difference between group 0 and any of the other exercise groups. There was no difference in mean predicted Lamminen-Mercuri score of thigh or leg muscles between groups (Figure 1).

Modeling using age and teenage exercise level could account for one third of the observed variability in Lamminen-Mercuri score in the whole leg (adjusted *R*^2^ = 0.33), while using age alone produced an *R*^2^ value of 0.14.

**Figure 2:**
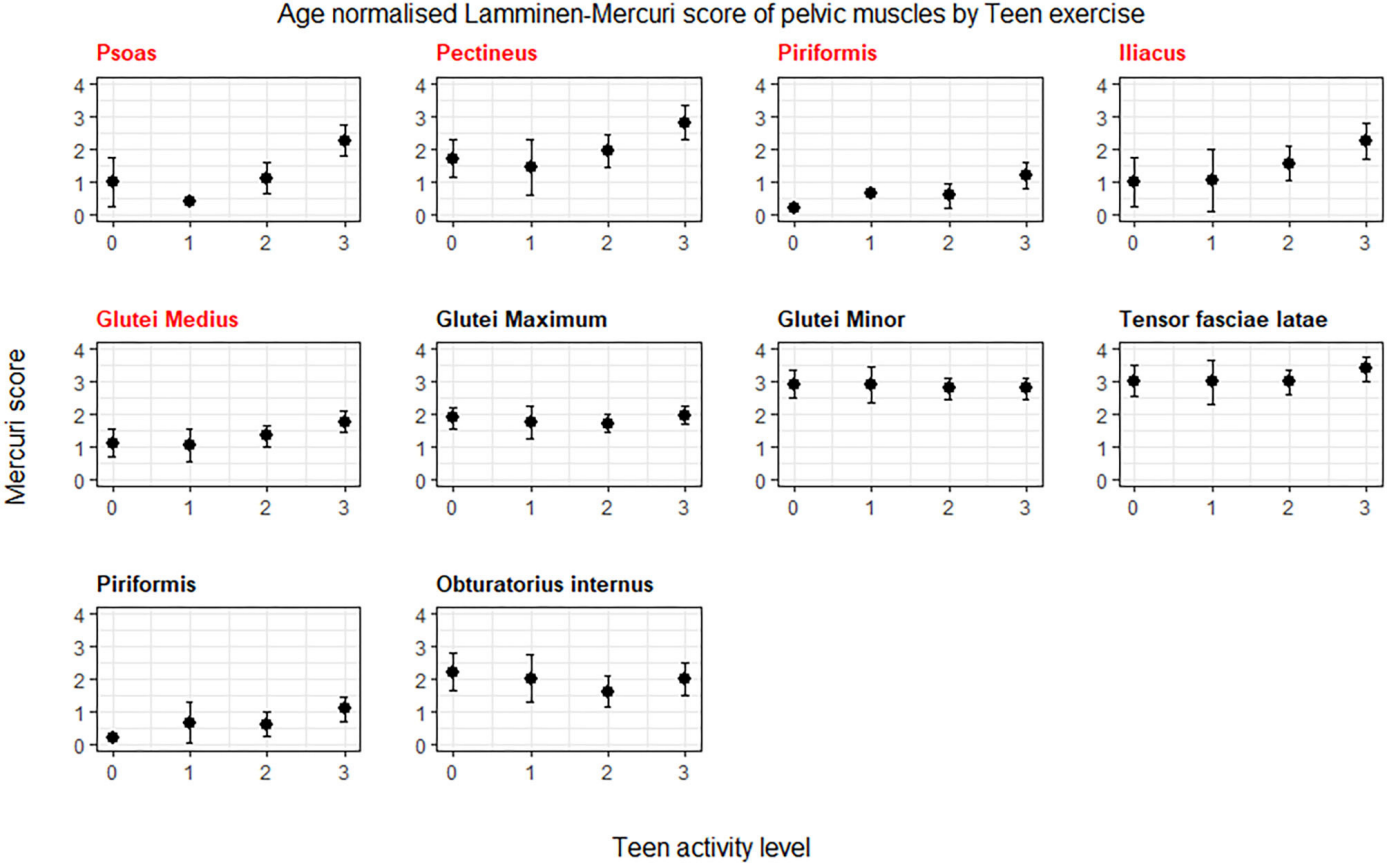
ANCOVA predicted Lamminen-Mercuri scores of the pelvis muscles, adjusted for age. Bars show the 95% confidence interval. Red text denotes groups showing significant differences on ANCOVA (*p* < 0.005). Patients are split into exercise groups 0, 1, 2, or 3.

The individual muscles in the pelvis showing a significant difference (*p* < 0.05) in age normalized Lamminen-Mercuri score between exercise groups were psoas, piriformis, pectineus, iliacus, and gluteus medius (Figure 2). However, after correction for multiple comparisons, this only remained significant for psoas (*p* = 0.0004), which showed a significantly higher predicted Lamminen-Mercuri score in exercise group 3 compared to groups 0 (*p* = 0.03), 1 (*p* = 0.0032), and 2 (*p* = 0.007).

**Figure 3:**
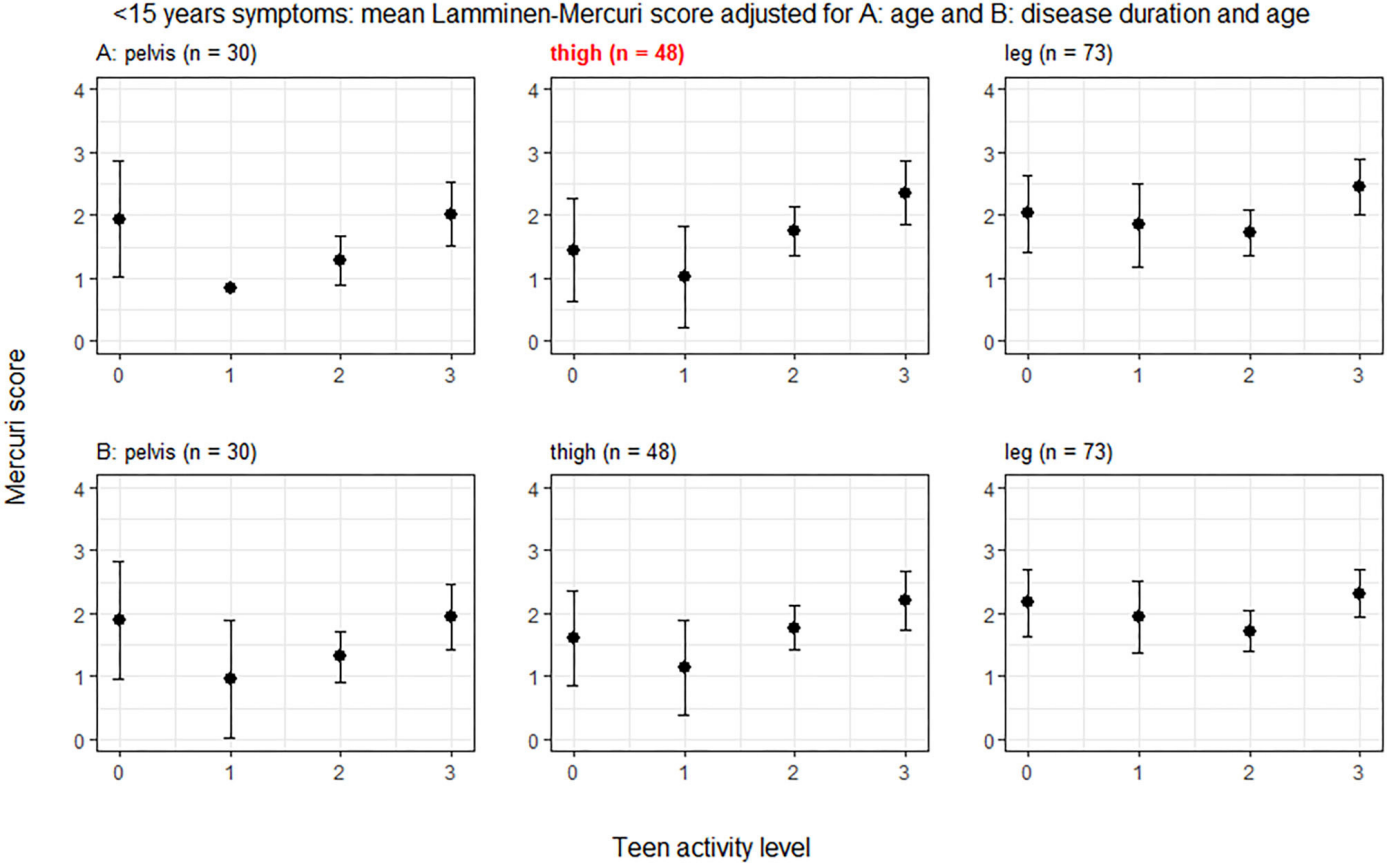
ANCOVA predicted Lamminen-Mercuri scores for each exercise group in those with <15 years of symptoms. The top row **(A)** shows predicted score in each exercise group (0, 1, 2, or 3) adjusted for age—i.e., as though all patients were the cohort mean age of 31 years. The bottom row **(B)** shows predicted score in each exercise group (0, 1, 2, or 3) adjusted for disease duration and age—i.e., as though all patients had an age of 31 years and the cohort mean symptom duration of 8 years. Bars show the 95% confidence interval. Red text denotes groups showing significant differences on ANCOVA (*p* < 0.005).

In the smaller subset of patients (*n* = 73) with symptoms for <15 years, there was a trend toward greater Lamminen-Mercuri score in groups 3 than 2 and in group 2 than 1 in the pelvis and thigh. However, there was no longer a significant difference in pelvic muscles by exercise group and although there was a difference between groups in the thigh muscles (*p* = 0.044), this did not remain after correction for multiple comparisons (Figure 3).

### Symptom Duration and Age Adjusted Mercuri Score

In ANCOVA of Lamminen-Mercuri score with symptom duration, age and exercise group as covariates, symptom duration was always a stronger predictor of Lamminen-Mercuri score than age and age was not an independent predictor of Lamminen-Mercuri score.

Symptom duration was a strong predictor of Lamminen-Mercuri score and modeling using symptom duration alone accounted for 38% of the variation in Lamminen-Mercuri score (adjusted *R*^2^ = 0.38) in the whole leg. Adding exercise group to the model improved the adjusted *R*^2^ value to 0.47, which was not bettered by the further addition of age to the model (adjusted *R*^2^ = 0.47). When accounting for symptom duration and age, there remained significant differences in mean predicted Lamminen-Mercuri score of the pelvic muscles (*p* = 0.0015) between exercise groups. Again, this was driven by a significantly higher predicted Lamminen-Mercuri score in exercise group 3, compared to group 2 (3 vs. 2, *p* = 0.0029) and group 1 (3 vs. 1, *p* = 0.0148). There was no significant difference between group 0 and any of the other exercise groups in the pelvis. There were no differences between exercise groups in thigh or leg muscle groups (Figure 1).

**Figure 4:**
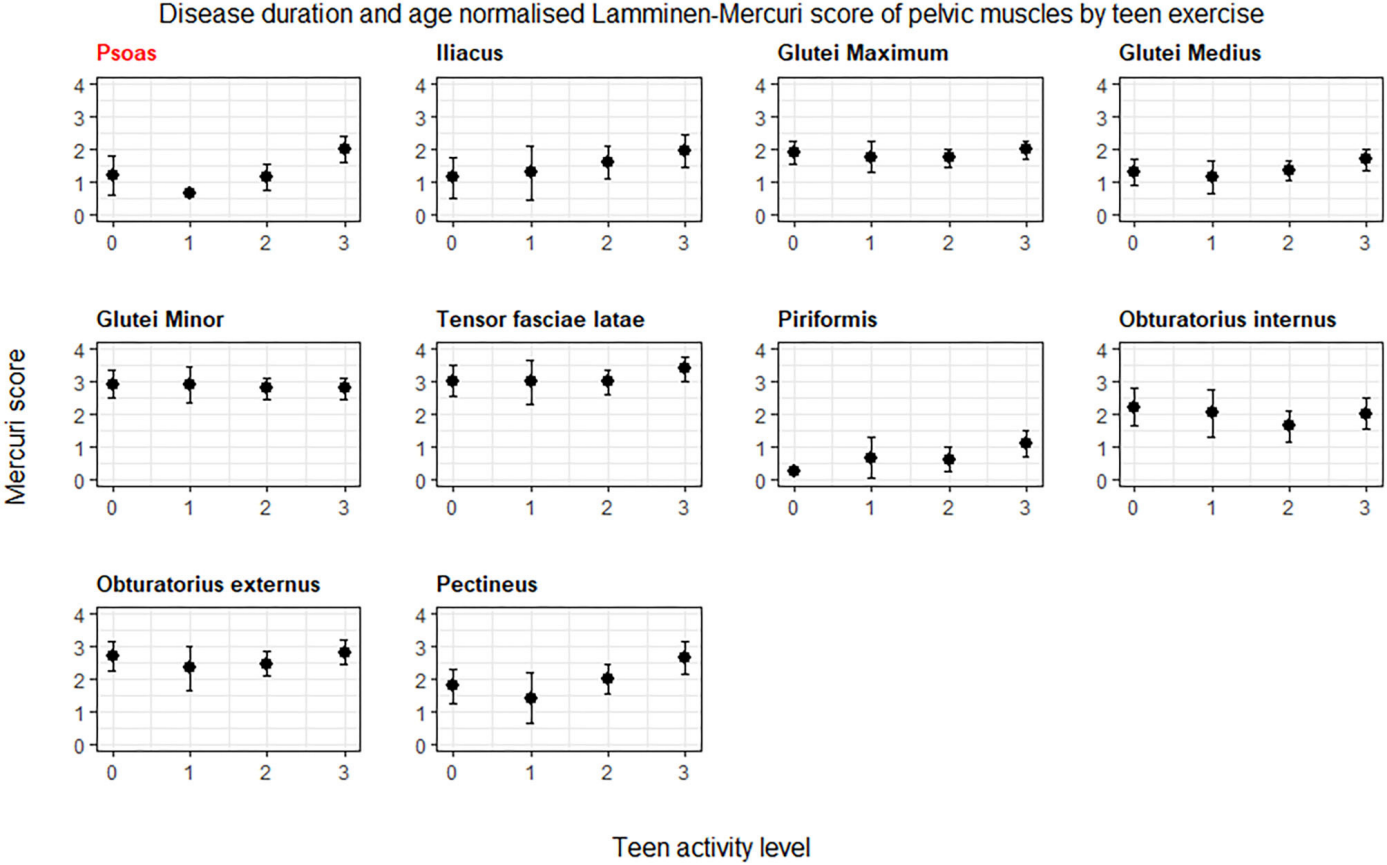
ANCOVA predicted Lamminen-Mercuri scores of the pelvis muscles, adjusted for age, and disease duration. Bars show the 95% confidence interval. Red text denotes groups showing significant differences on ANCOVA (*p* < 0.005). Patients are split into exercise groups 0, 1, 2, or 3.

**Figure 5:**
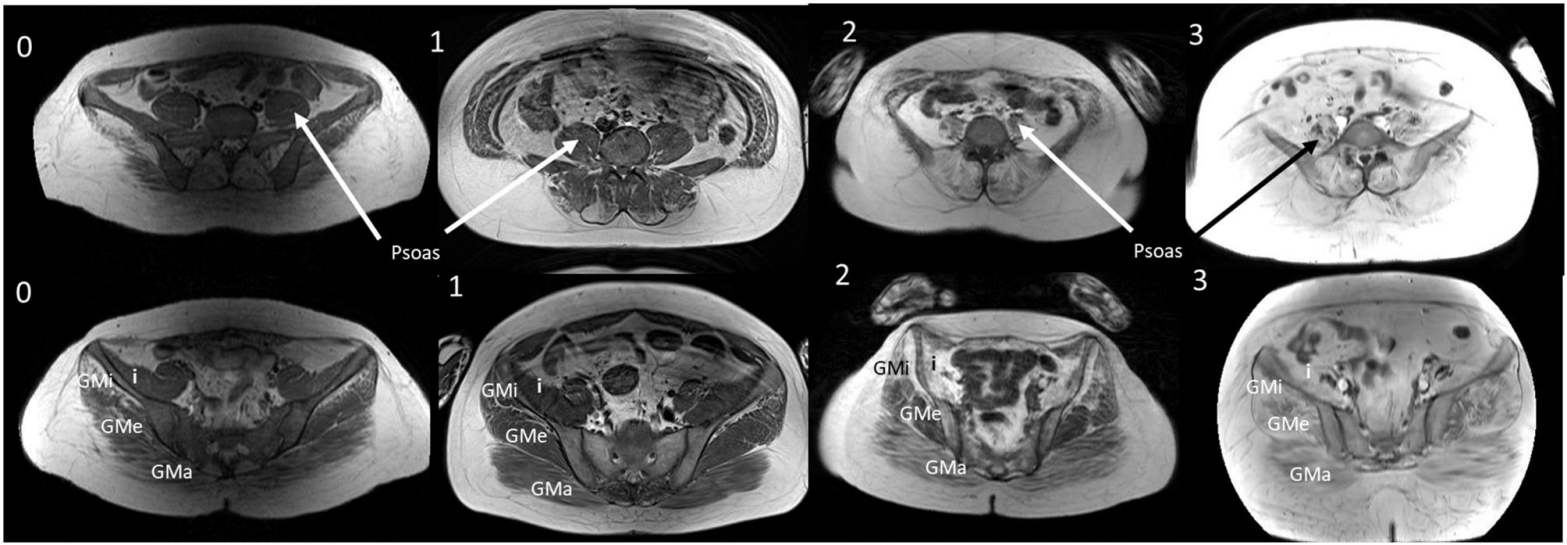
T1W MRI images from age and duration matched patients. T1W MRI images of the lower abdomen and pelvisfor a representative patient from each exercise group (0, 1, 2, and 3) of a similar age and symptom duration. GMa, gluteus maximus; GMe, gluteus medius; GMi, gluteus minimus; I, Iliacus. Representative patients from groups 0, 2, and 3 were all aged 36 years with symptom duration 18 or 19 years at the time of the MRI. Group 1 patient was aged 33 years with a symptom duration of 21 years.

The muscle in the pelvis showing the greatest difference in age and symptom duration adjusted Lamminen-Mercuri score between exercise groups remained psoas (*p* = 0.016) (Figure 4, example MRI images in Figure 5). However, differences between groups in psoas Lamminen-Mercuri score were not significant on ANCOVA after correction for multiple comparisons.

In the smaller subset of patients with symptoms for <15 years, there was no longer a significant difference between exercise groups in any of the muscle groups.

## Discussion

We have demonstrated an association between intensive teenage exercise and a subsequent more severely affected MRI appearance of pelvic musculature in adult patients with dysferlinopathy, long after their teenage years. Psoas, pectineus, and piriformis were the most differentially affected muscles. Patients who performed intensive exercise as teenagers generally demonstrated a higher (worse) Lamminen-Mercuri score at a given age and disease duration, suggesting a more rapid progression of fat replacement from symptom onset.

We did not demonstrate any association between exercise group and the muscle groups of the thigh or distal leg in the overall group, but there was a trend toward a higher Lamminen-Mercuri score in the thigh muscle of intensive exercisers with <15 years symptom duration. The thigh and leg muscles generally have a higher Lamminen-Mercuri score in this cohort than the muscles of pelvis (9), suggesting earlier or more significant involvement of these muscles in the disease process. It may be that no difference was demonstrated between exercise groups in the thigh and distal leg overall because too many patients are already at a stage where these muscles are severely affected, essentially reaching a ceiling of the maximum Lamminen-Mercuri score of 4 relatively early in the disease process. The finding of potential differences in the thigh muscles in less advanced disease suggests that there may have been differences in all muscles at some point in progression of the disease.

In earlier work, we showed that symptom onset and age of part and full time wheelchair use is significantly earlier in intensive than moderate exercisers (4). While this MRI study adds objective evidence to this observation, we did not observe the previously described “dose” type of association with exercise—with non and very light exercisers (groups 0 and 1) having a significantly later age of onset and wheelchair requirement than those in group 2. We had anticipated that when investigating the MRI, this may translate to differences in Lamminen-Mercuri score between exercise group 0, group 2, and group 3. However, this was not seen and the key differences in Lamminen-Mercuri score were between exercise groups 2 and 3 and not group 0. There are several possible explanations for this. Firstly, this may be related to the possible ceiling effect of the Lamminen-Mercuri score, as patients in group 0 were significantly older and had had symptoms for significantly longer than patients in group 2 and 3, making them more likely to have reached this plateau. It is also possible that, the more time that intervenes between teenage years and subsequent assessment the less marked the effect of exercise on imaging results—this would again dilute any effect in the older group 0. Finally, while the majority of non-exercisers had a relatively late onset of symptoms, some had a younger onset and probably did not exercise precisely because they had already started to experience symptoms. The muscle MRI of these more severely affected patients may “skew” the results of the non-exercising group 0.

The method used here to quantify the “amount” of exercise performed used the relative metabolic equivalents of the sports performed, giving an estimate of the intensity of the exercise. However, research in mice suggests that it may be the type of muscle contraction, rather than the metabolic cost, which determines the effect on dysferlin deficient muscle (5, 6). Unfortunately we were not able to assess this here because, as anticipated in an observational study, none of our participants had performed exclusively concentric exercise (such as swimming) without also reporting frequent eccentric sports (such as running).

We have identified specific muscles in the pelvis that were more affected in intensive exercisers in this cohort. This raises the question of why these particular muscles are affected while others are not. This may in part be due to differences only being observable in less affected muscle, as discussed above. However, there were some muscles which are not extensively involved across exercise groups (such as gluteus maximum) and yet still do not demonstrate differential involvement. It seems reasonable that sports that preferentially use specific muscles could affect these muscles more than others, and as many intensive sports involve a significant amount of running, intensive exercisers may see proportionally greater damage in muscles involved heavily in running—such as psoas (11, 12). We did attempt to review this in our cohort, comparing Lamminen-Mercuri scores for individual muscles between patients who reported different activities (e.g., predominantly running vs. predominantly swimming). We did not find differential Lamminen-Mercuri scores between the most frequently performed activities, however this analysis is likely significantly confounded by the multiple activities performed by each patient.

Muscle MRI offers the possibility to analyse the impact of exercise in muscle structure. In our opinion, the results of this study should encourage further research in muscle MRI biomarkers in dysferlinopathy patients performing different types of exercise. Muscle MRI sequences that could be helpful include but are not limited to STIR, T2 imaging, sodium MRI, and P31-spectroscopy (13–15). All of these previous imaging sequences have identified early changes in muscle structure, that in the case of dysferlinopathy patients, could help to elucidate which program of exercise is less harmful for the skeletal muscles.

This analysis confirms that T1W imaging demonstrates more severe fat replacement in muscles of patients with dysferlinopathy who performed intensive, rather than moderate, exercise as teenagers. This adds pathological evidence to the previous report linking symptom onset and earlier wheelchair use to intensive teenage exercise. For patients who receive a diagnosis before symptom onset, such as through family screening or detection of high CK, this evidence would support a recommendation to avoid very intensive exercise regimens before symptom onset, while maintaining a healthy lifestyle.

## Data Availability Statement

The raw data supporting the conclusions of this article will be made available on reasonable request to the study steering group. Requests should be directed to Professor Volker Straub at volker.straub@newcastle.ac.uk.

## Ethics Statement

The studies involving human participants were reviewed and approved by local ethics committees in each country involved in the study: Comité Etico de investigacion con médicamentos de la Fundacio de Gestio Sanitaria de la Santa Creu I Sant Pau, Barcelona, Spain; The Carolinas HealthCare System Institutional Review Board Federal-Wide Assurance, Charlotte, United States; IRB at Nationwide Children's Hospital, Columbus, United States; Le Comité de Protection des Personnes Sud-Méditerranée, Marseille and Paris, France; Ethikkomission bei de LMU Munchen, Munich, Germany; Stanford University IRB, Panel on Human Subjects, Panel 7, Stanford, United States; The Washington University in St. Louis Institutional Review Board, St Louis, United States; The Medical Ethics Committee of the NCNP, Tokyo, Japan; Children's National Medical Center Institutional Review Board (Children's National IRB), Washing DC, United States; Newcastle and North Tyneside 2 medical ethics committee, Newcastle, United Kingdom; El comité de Etica de a investigacion de Centro H.U Virgen del Rocio de Sevilla, Seville, Spain; REGIONE VENETO AZIENDA OSPEDALIERA DI PADOVA Comitato Etico per la Sperimentazione, Padova, Italy; Children's Hospitals Network Human Research Ethics Committee, Sydney, Australia; North East—Newcastle and North Tyneside Health Research Authority research ethics committee, Newcastle, United Kingdom. The patients/participants provided their written informed consent to participate in this study.

## Author Contributions

UM contributed to data acquisition, conception and study design, analysis of data, and preparation of the manuscript. MaJ contributed to review of statistical analysis and drafting. RF-T, JL, FS, MeJ, AM, LR, PC, AB, JD, KJ, DB-G, ES-C, AP, MW, CP, TS, MM-Y, EB, EP, JM, and KB contributed to acquisition and analysis of data and drafting. VS contributed to conception and study design, acquisition and analysis of data, and drafting. JD-M contributed to conception and study design, acquisition and analysis of data, drafting, and final sign of corresponding author. All authors contributed to the article and approved the submitted version.

## Conflict of Interest

UM, MJa, LR, AB, and AP reports the grant from the Jain Foundation. JD reports the grant from the Jain Foundation, personal fees from Biogen, Ionis, Avexis, Roche, Sarepta, Sanofi, Genzyme, Scholar Rock, Pfizer plus patents from Athena Diagnostics. DB-G reports membership of the Gene Therapy Network (Avexis). MW reports advisory board membership for Avexis, Biogen, Novartis, Roche, Santhera, Sarepta, PTC Therapeutics, Ultragenyx, Wave Sciences, plus personal fees from Novartis, Biogen, Ultragenyx, Santhera, PTC Therapeutics, Ask Bio, Audentes Therapeutics, Fulcrum Therapeutics, GIG Consul, Guidepoint Global, Novartis, PTC, Gruenthal Pharma. EP reports grants, personal fees, and non-financial support from Santhera, personal fees, and non-financial support from Sarepta, Personal fees, and non-financial support from PTC pharmaceuticals all outside this submitted work. VS reports the Jain Foundation grant and other grants and personal fees from Sarepta Therapeutics. The remaining authors declare that the research was conducted in the absence of any commercial or financial relationships that could be construed as a potential conflict of interest.
