# Supplementary material for: Intensive Teenage Activity Is Associated With Greater Muscle Hyperintensity on T1W Magnetic Resonance Imaging in Adults With Dysferlinopathy
Source: Front Neurol. 2020 Dec 16;11:613446. doi: 10.3389/fneur.2020.613446 (PMC7773023; doi:10.3389/fneur.2020.613446)
Supplement: Supplementary file 1 [file Data_Sheet_1.PDF]

Exercise question from screening questionnaire in COS1

Did the patient frequently participate in a sports, dance or other physical activity *before the first* muscle problems presented?

☐ no
☐ the patient does not have any muscle symptoms
☐ unknown

☐ yes

If yes, *please provide details about the type of past sports, dance or other physical activities, the frequency of their performance, how many years the patient participated in them (or if the patient continues to do the activities) and at what level, and indicate at what age muscle problems began to interfere with each activity (if applicable). Several answers (activities) are possible.*

sports/dance /other physical activity	seasonal frequency *(please tick one option)*	frequency *(please tick one option)*	from what age *(if remembers)*	to what age *(if remembers)* or continuing	level of performance *(please tick one option)*	age at which muscle problems began to interfere with activity *(if applicable)*
☐ running	☐ throughout the year<br>☐ seasonal only	☐ daily<br>☐ multiple times a week<br>☐ once a week<br>☐ monthly<br>☐ occasionally			☐ worse than average<br>☐ average<br>☐ better than average<br>☐ national/regional representation	
☐ cycling	☐ throughout the year<br>☐ seasonal only	☐ daily<br>☐ multiple times a week<br>☐ once a week<br>☐ monthly<br>☐ occasionally			☐ worse than average<br>☐ average<br>☐ better than average<br>☐ national/regional representation	
☐ swimming	☐ throughout the year<br>☐ seasonal only	☐ daily<br>☐ multiple times a week<br>☐ once a week<br>☐ monthly<br>☐ occasionally			☐ worse than average<br>☐ average<br>☐ better than average<br>☐ national/regional representation	
☐ soccer	☐ throughout the year<br>☐ seasonal only	☐ daily<br>☐ multiple times a week<br>☐ once a week<br>☐ monthly<br>☐ occasionally			☐ worse than average<br>☐ average<br>☐ better than average<br>☐ national/regional representation	

☐ American football	☐ throughout the year<br><br>☐ seasonal only	☐ daily<br>☐ multiple times a week<br>☐ once a week<br>☐ monthly<br>☐ occasionally			☐ worse than average<br>☐ average<br>☐ better than average<br>☐ national/regional representation	
☐ ballet	☐ throughout the year<br><br>☐ seasonal only	☐ daily<br>☐ multiple times a week<br>☐ once a week<br>☐ monthly<br>☐ occasionally			☐ worse than average<br>☐ average<br>☐ better than average<br>☐ national/regional representation	
☐ tennis	☐ throughout the year<br><br>☐ seasonal only	☐ daily<br>☐ multiple times a week<br>☐ once a week<br>☐ monthly<br>☐ occasionally			☐ worse than average<br>☐ average<br>☐ better than average<br>☐ national/regional representation	
☐ figure skating	☐ throughout the year<br><br>☐ seasonal only	☐ daily<br>☐ multiple times a week<br>☐ once a week<br>☐ monthly<br>☐ occasionally			☐ worse than average<br>☐ average<br>☐ better than average<br>☐ national/regional representation	
☐ hockey	☐ throughout the year<br><br>☐ seasonal only	☐ daily<br>☐ multiple times a week<br>☐ once a week<br>☐ monthly<br>☐ occasionally			☐ worse than average<br>☐ average<br>☐ better than average<br>☐ national/regional representation	
☐ basketball	☐ throughout the year<br><br>☐ seasonal only	☐ daily<br>☐ multiple times a week<br>☐ once a week<br>☐ monthly<br>☐ occasionally			☐ worse than average<br>☐ average<br>☐ better than average<br>☐ national/regional representation	
☐ volleyball	☐ throughout the year<br><br>☐ seasonal only	☐ daily<br>☐ multiple times a week<br>☐ once a week<br>☐ monthly<br>☐ occasionally			☐ worse than average<br>☐ average<br>☐ better than average<br>☐ national/regional representation	
☐ handball	☐ throughout the year<br><br>☐ seasonal only	☐ daily<br>☐ multiple times a week<br>☐ once a week<br>☐ monthly<br>☐ occasionally			☐ worse than average<br>☐ average<br>☐ better than average<br>☐ national/regional representation	
☐ baseball	☐ throughout the year<br><br>☐ seasonal only	☐ daily<br>☐ multiple times a week<br>☐ once a week<br>☐ monthly<br>☐ occasionally			☐ worse than average<br>☐ average<br>☐ better than average<br>☐ national/regional representation	
☐ cricket	☐ throughout the year<br><br>☐ seasonal only	☐ daily<br>☐ multiple times a week<br>☐ once a week<br>☐ monthly<br>☐ occasionally			☐ worse than average<br>☐ average<br>☐ better than average<br>☐ national/regional representation	

☐ rugby	☐ throughout the year<br><br>☐ seasonal only	☐ daily<br>☐ multiple times a week<br>☐ once a week<br>☐ monthly<br>☐ occasionally			☐ worse than average<br>☐ average<br>☐ better than average<br>☐ national/regional representation	
☐ golf	☐ throughout the year<br><br>☐ seasonal only	☐ daily<br>☐ multiple times a week<br>☐ once a week<br>☐ monthly<br>☐ occasionally			☐ worse than average<br>☐ average<br>☐ better than average<br>☐ national/regional representation	
☐ dancing	☐ throughout the year<br><br>☐ seasonal only	☐ daily<br>☐ multiple times a week<br>☐ once a week<br>☐ monthly<br>☐ occasionally			☐ worse than average<br>☐ average<br>☐ better than average<br>☐ national/regional representation	
☐ athletics (track and field)	☐ throughout the year<br><br>☐ seasonal only	☐ daily<br>☐ multiple times a week<br>☐ once a week<br>☐ monthly<br>☐ occasionally			☐ worse than average<br>☐ average<br>☐ better than average<br>☐ national/regional representation	
☐ step aerobics	☐ throughout the year<br><br>☐ seasonal only	☐ daily<br>☐ multiple times a week<br>☐ once a week<br>☐ monthly<br>☐ occasionally			☐ worse than average<br>☐ average<br>☐ better than average<br>☐ national/regional representation	
☐ ___________	☐ throughout the year<br><br>☐ seasonal only	☐ daily<br>☐ multiple times a week<br>☐ once a week<br>☐ monthly<br>☐ occasionally			☐ worse than average<br>☐ average<br>☐ better than average<br>☐ national/regional representation	
